# Potassium-induced plant resistance against soybean cyst nematode via root exudation of phenolic acids and plant pathogen-related genes

**DOI:** 10.1371/journal.pone.0200903

**Published:** 2018-07-30

**Authors:** Xiang Gao, Shuxiang Zhang, Xiujuan Zhao, Qihua Wu

**Affiliations:** Institute of Agricultural Resources and Regional Planning, Chinese Academy of Agricultural Sciences, National Engineering Laboratory for Improving Quality of Arable Land, Beijing, P. R. China; Estacion Experimental del Zaidin, SPAIN

## Abstract

Soybean cyst nematode (SCN) is a severe soil borne disease. The control of this disease is still a worldwide problem in agriculture. In this study, we found that application of potassium (K) fertilizer could decrease the occurrence of SCN at two field sites. Furthermore, the application of K could suppress *Heterodera glycines* with the activation of Phenylalanine Ammonia Lyase (*PAL)* and Polyphenol Oxidase (*PPO)* expression via pot experiments in a greenhouse. The release of cinnamic, ferulic and salicylic acids was significantly enhanced by K application of 3 mM, and each of three acids can dramatically constrain *Heterodera glycines* in vitro. This research indicated that K induce multiple mechanisms to improve the resistance of soybean against SCN and provide a new strategy to control SCN in fields with nutrient application.

## Introduction

Soybean (*Glycine max* (L.) Merr.) accounts for 7% of the total crop cultivation area and is one of the economically important crops in China. In recent decades, the yield of soybean has been seriously affected by soil borne diseases [1]. Soybean cyst nematode (SCN), caused by *Heterodera glycines* Ichinohe (*H*. *glycines*), is one of the most serious soil borne diseases [1,2]. Soybean cyst nematode is a microscopic roundworm that feeds on the roots of the soybean plant [3]. The disease generally reduces soybean yield from 20–50% [4]. Normally, nematicide and soil fumigation are proposed to control SCN [5,6]. However, such treatments seriously affect the ecological environment [7,8]. Therefore, simple tactics with fewer environmental risks have been sought to prevent SCN. It has been evidenced that the application of chemical fertilizers at optimum levels could reduce the risk of soil borne diseases of crops [9,10].

Potassium (K) is one of the essential plant nutrients that is believed to influence crop metabolism and development as well as yield and even affects the occurrence of plant disease [10–12]. Many studies have proven that the application of K fertilizer can beneficially protect plants against various pathogens [11]. For instance, the application of K can significantly prevent sheath rot disease, and similarly, it can reduce the risk of anthracnose disease in dogwood leaves [13].

Potassium nutrition can induce a plant’s resistance against diseases mainly through increasing nutrient resources, changing the primary metabolism and hormonal responses, etc. [13,14]. Many studies have found that application of K fertilizer can enormously reduce the incidence of crop stalk, leaf or root diseases, which has been attributed to K nutrition of crop tissue, which promotes the activities of enzymes and induces abundant natural compounds [11,13,15]. These results have been well documented by the main potential mechanisms to control plant diseases [10,15]. For instance, the application of KCl in the foliage can prevent wheat powdery mildew [10]. Similarly, the application of K fertilizer in dogwood leaves can also reduce the risk of anthracnose attack [12].

However, until now, few studies have researched K fertilizer in controlling SCN in the field and the related mechanisms. In this study, we found that optimizing application of K fertilizer could decrease SCN disease in the field. Therefore, we hypothesize that the optimized application of K fertilizer would improve plant physiological capacity against cyst nematode. Then, a sand culture in a greenhouse was conducted to elucidate the effect of various K level treatments on SCN. Root exudation of soybean was collected to test phenolic acids, and two pathogen-related gene expression, namely PAL and PPO. Simultaneously, the mortality rate of *H*. *glycines* from the root system was tested under various phenolic acid treatments *in vitro*. Our research have demonstrated that the application of K fertilizer could induce root exudation of phenolic acids and enhance plant pathogen-related genes express to control SCN.

## Materials and methods

1. The Zhangjiachi (E124.30^o^, N42.94^o^) and Changling (E123.98^o^, N44.15 ^o^) field site is the experimental base of the Chinese Academy of Agricultural Sciences, Institute of Agricultural Resources and Regional Planning. Therefore, the authority that issued the permit for this location is Chinese Academy of Agricultural Sciences.
2. No specific permissions were required for these locations. I am here to confirm that the field studies did not involve endangered or protected species.

### Field trials

Field experiments were carried out at soybean field sites naturally infested by SCN at Zhangjiachi and Changling in Liaoning and Jilin Provinces, respectively, China, for two consecutive years in 2012 and 2013. The soil chemical characteristics were as follows: 1) for Zhangjiachi: pH value, 7.8; organic matter, 21.5 g kg^-1^; available P, 11.1 mg P kg^-1^; available N, 102.8 mg N kg^-1^; and available K, 83.7 mg K kg^-1^; and 2) for Changling: pH value, 6.5; organic matter, 15.2 g kg^-1^; available P, 14.1 mg P kg^-1^; available N, 144.9 mg N kg^-1^; and available K, 92.6 mg K kg^-1^. The soybean variety FS25 was susceptible to SCN and used in the field experiments.

To test the suppression effect of the K fertilizer on SCN, three treatments of K fertilizer were designed as follows: (1) K0 included no K fertilizer application; (2) K60 applied K at a rate of 60 kg K_2_O ha^-1^; and (3) K120 applied K at a rate of 120 kg K_2_O ha^-1^. The K fertilizer of 60 and 120 kg ha^-1^ represented medium and high K concentrations in field trials, respectively. Urea and P_2_O_5_ were applied to all plots as 80 kg N ha^-1^ and 60 kg P ha^-1^ for supplementing N and P supplies, respectively. Each treatment had 4 replicates, which covered at least a 60 m^2^ plot^-1^ planting area in a completely randomized design.

At harvest, 50 plants were selected randomly from each plot for measured yield and analyzed for the disease index of SCN that was recorded for each plant on a 0–5 scale, where 0 = no cyst nematode on the root; 1 = cyst nematode covers 1–20% of the root system; 2 = cyst nematode covers 21–40% of the root system; 3 = cyst nematode covers 41–60% of the root system; 4 = cyst nematode covers 61–80% of the root system; and 5 = plant is dead and cyst nematode covers over 80% of the root system. The disease index (DI) for each plot was calculated by the following equation: DI (%) = {[(n_1_×1)+(n_2_×2)+(n_3_×3)+…+(n_N_×N)] /[N×(n_1_+n_2_+n_3_…+n_N_)]} ×100, where n_1_…n_N_ is the number of cyst nematodes in each of the respective disease categories and N is the highest scoring of the disease [16]. Representative plant samples collected from each plot were analyzed for their content of total phenol and K concentration. Simultaneously, 100 g of rhizosphere soil of the soybean was collected to calculate the number of cyst nematodes.

### Pot sand culture

Pot culture experiments were conducted in the greenhouse at the Chinese Academy of Agricultural Sciences (CAAS) from March to June 2016 in Beijing, China. Seeds of FS25 were sown in sand. The seedlings were grown under natural light at 30/22 (day/night) ^o^C with a relative humidity of 70–90%. The pathogen *H*. *glycines* was isolated from the diseased soybean roots and used for inoculation. The inoculum density was determined by hatching a second-stage juvenile (J2) of *H*. *glycines* at 3000 units per pot.

The sand culture experiment was as follows: (1) The four K levels were K0 (0 mM K); K1 (1 mM K); K3 (3 mM K); and K6 (6 mM K). The K1, K3 and K6 represented low, medium and high K concentrations in sand cultures, respectively. (2) The soybean plants were inoculated with *H*. *glycines* (+SCN) or sterilized media as a control (-SCN) when the soybean was at the V5 growth stage. The total number of treatments performed was 8, and each replicate had 16 pots with three plants in each of the pots. Plants were watered daily using modified 1/2 strength Hoagland nutrient solution with spiking of the four levels of K. After 30 days of inoculation of *H*. *glycines*, the number of cyst nematodes on the soybean roots was measured. Meanwhile, plant samples were collected to determine the biomass, total phenol content and K concentration.

### Assay of PR-gene and enzyme activity

Expression levels of selected pathogen-related (PR) genes of soybean in response to K and inoculation of *H*. *glycines* were quantified by RT-PCR as referred to by Gao et al. [16]. The specific primer sequences were *PAL* (X52953), forward/reverse primers, GTGCAAGGGCTGCTTATG, CCCAGTCCCTAATTCCTCTC, *PPO* (EF158428) GGGTTGGTGCTGCTGATAAG, CGATCCGAGTTCGTGTGATG. The activities of polyphenol oxidase (PPO) and phenylalanine ammonia-lyase (PAL) were determined as described by Song et al. [17]. PAL is related to phenol metabolism, which is the key enzyme of phenylpropane metabolism and synthesis of lignin [17,18]. And PPO is the key enzyme for the oxidation of phenolic substances, when the plants are infected by the pathogens, the PPO can promote the synthesis of phenolic compounds and impede the invasion of pathogens [17,18]. We have designed 16 pots for each treatment and randomly selected 9 pots for sampling on RT-PCR analysis. We set up 3 pots as one biological replicate and took one soybean root in each pot, and then 3 soybean roots have been mixed for extraction RNA. Three independent biological replicates for each treatment were determined. The detailed calculation methods can be found in S1 File.

### Analysis of phenolic acid varieties in soybean root exudates

The root exudates were collected at the soybean florescence stage (R2 growth stage) when the soybean plants had strong allelopathic potentials [18]. Three soybean seedlings were gently taken out of the pot and washed with deionized water several times. The cleaned roots were submerged in a plastic cup containing 500 mL of 0.5 μM CaCl_2_ for 6 hours to collect exudates. The cup was covered by a black lid to avoid contamination and light. Root exudates were filtered through a 0.22-μm filter and then lyophilized and stored at -80 ^o^C for subsequent analysis. The lyophilized powder of the soybean root exudates was analyzed for phenolic acid variety and bioassay.

Phenolic acids from the root exudates were identified using a high-performance liquid chromatography (HPLC) system (Agilent 1200, Germany). Eight types of phenolic acids, namely gallic acid, *p-*coumaric acid, phthalic acid, vanillic acid, syringic acid, ferulic acid, salicylic acid and cinnamic acid, were used as standard phenolic compounds. Analytical conditions and methods were applied according to the manufacturer’s instructions as described by Ling et al. [19].

### Bioassay on *Heterodera glycine in vitro*

A bioassay on the mortality rate of *H*. *glycines* was conducted by adding 5 mL of root exudates to a petri dish. Plates were incubated at 28 ^o^C in the dark. The rate of mortality of *H*. *glycines* was determined in three days. Nematode mortality was determined as the percentage of dead nematode on total number of tested nematodes. 100 nematodes were placed in each petri dish and 4 replicates were set up.

According to the results from HPLC analysis, the dominant phenolic acids, including ferulic, vanillic, cinnamic and salicylic acids, were subsequently exogenously applied with phenolic acids (Sigma, USA) for allelopathic assay. Four treatments with different concentration levels (0, 20, 40, and 60 mg L^-1^) were applied in the petri dish to test the mortality rate of *H*. *glycines* as previously described.

### Data analysis

The data obtained from the experiments were statistically analyzed by two-way ANOVA using Excel 2007 software (Microsoft Corporation, 1985–2007) and SAS 9.1 (SAS Inc., Cary, NC, USA).

## Results

### Effects of K fertilizer on SCN disease in field trials

As shown in Table 1, the application of K fertilizer significantly reduced DI and cyst nematode numbers in field trials at the Zhangjiachi and Changling sites for two consecutive years. Compared to the K0 treatment at the Zhangjiachi field site, the DI was reduced by 22% and 26% in 2012 and 2013, respectively, under K60 treatment. Similarly, at the Changling field site, the DI was reduced by 31% and 25%, respectively, for the two years under K60 treatment. However, the differences between the DIs of the K120 and K60 treatments were not significant (Table 1).

**Table 1:** Effect of different K application levels on the disease index (DI) of soybean cyst nematode and cyst nematode number (CNN) in the two field trials over two years.

Year	K treatment	Zhangjiachi		Changling	
		DI	CNN	DI	CNN
	K0	85±4a	76±7a	52±3a	52±4a
2012	K60	66±2b	50±6b	36±2b	33±4b
	K120	69±2b	56±3b	38±2b	37±6b
	K0	77±2a	82±5a	56±3a	66±4a
2013	K60	57±3b	61±6b	42±4b	48±6b
	K120	62±3b	63±6b	42±3b	53±5b

Note: Disease index and cyst nematode number were measured as described in the Materials and Methods. K0, no K application; K60, 60 kg ha^-1^ K; K120, 120 kg ha^-1^ K. All of the data are the means ± SE (n = 4). The different letters after the numbers in the same column for the same trait in the same year indicate significant differences (*P<0*.*05*) by Duncan’s t-tests.

Correspondingly, the numbers of cyst nematodes of the K treatments (including K60 and K120) were significantly lower than in the K0 treatments at both field sites (Table 1). The cyst nematode numbers under K60 treatment were 34% and 26% lower compared to the K0 treatment at the Zhangjiachi field site in 2012 and 2013, respectively. The numbers of cyst nematodes under K60 treatment at the Changling field site were 37% and 27% lower in 2012 and 2013, respectively, compared to the K0 treatment (Table 1). Similar to the DI of SCN, the difference between the cyst nematode numbers of the K60 and K120 treatments was not significant.

### Yield, content of K concentration and phenols of soybean in the field experiments

The application of K fertilizer conspicuously increased the yield, content of total phenols and K concentration of soybean (Table 2). In the two field experiments, with the increase in K fertilizer, there was a significant increase in the yield of soybean. The K60 treatment yield was 25% and 30% greater when compared to the K0 treatment in 2012 and 2013 at Zhangjiachi, respectively. There was a similar trend in the soybean yield at the Changling field site. However, there was no significant difference between the K120 and K60 treatments at these two field sites.

**Table 2:** Effect of the different K application rates on the yield, K concentration and total phenol content of soybean plants in two field trials over two years.

Year	K treatment	Zhangjiachi			Changling		
		Yield (kg ha^-1^)	K (%)	Phenols (mg g^-1^)	Yield (kg ha^-1^)	K (%)	Phenols (mg g^-1^)
	K0	1338±105b	0.80±0.04b	1.12±0.05b	1505±64b	0.94±0.02c	0.94±0.11b
2012	K60	1671±94a	1.01±0.08a	1.53±0.12a	2015±135a	1.28±0.08b	1.33±0.10a
	K120	1664±93a	1.14±0.06a	1.72±0.16a	1929±122a	1.59±0.07a	1.42±0.09a
	K0	1534±88b	0.84±0.07c	1.10±0.08b	1409±58b	0.97±0.08c	1.02±0.13c
2013	K60	1990±98a	1.13±0.01b	1.83±0.08a	1853±65a	1.30±0.05b	1.32±0.06b
	K120	1867±87a	1.27±0.07a	1.98±0.08a	1916±132a	1.67±0.07a	1.60±0.13a

Note: K0, no K application; K60, 60 kg ha^-1^ K; K120, 120 kg ha^-1^ K. All of the data are the means ± SE (n = 4). The different letters after the numbers in the same column for the same trait in the same year indicate significant differences (*P<0*.*05*) by Duncan’s t-tests.

Regarding K concentration, K0 treatment led to the lowest K concentration in soybean plants. Simultaneously, K120 treatment resulted in the highest K concentration in soybean plants (Table 2). Regarding the content of total phenols, with an increase in K fertilizer, the content of total phenols improved. For the K120 treatment, there was a significant increase of 54% and 80% compared to K0, at the Zhangjiachi field site in 2012 and 2013, respectively. This indicated that the application of K fertilizer not only affected SCN but also interacted with the phenol content in the field trials.

### Correlation analysis between DI and related physiological parameters

**Fig 1:**
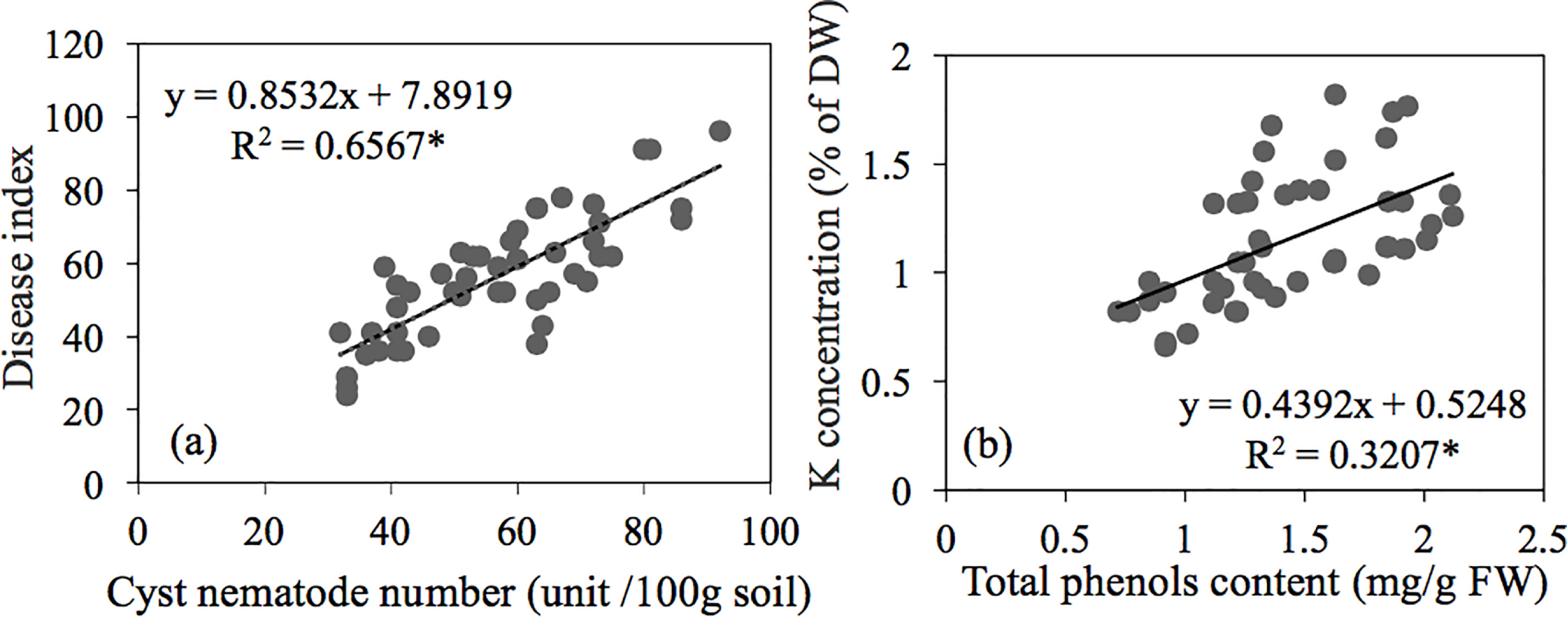
The correlation coefficient analysis of the two field trials over two years. (a) between disease index of soybean cyst nematode and cyst nematode number in rhizosphere soil parameter, and (b) between K concentration and total phenol content. For correlation coefficient analysis: *R*^2^ value *: significant at *P*<0.05, n = 48.

The DI of SCN and the cyst nematode number in the rhizosphere soil, K concentration and total phenol content of plants were used to conduct a correlation analysis under field experimental conditions (Fig 1). The results showed that the two sets of data were significantly correlated and that the *R*^2^ values reached up to 0.6567 and 0.3207, respectively. This result suggested that the numbers of cyst nematodes affected the degree of disease occurrence, while the K inputs significantly contributed to the synthesis of phenols.

### Severity of SCN in the sand culture experiment

**Fig 2:**
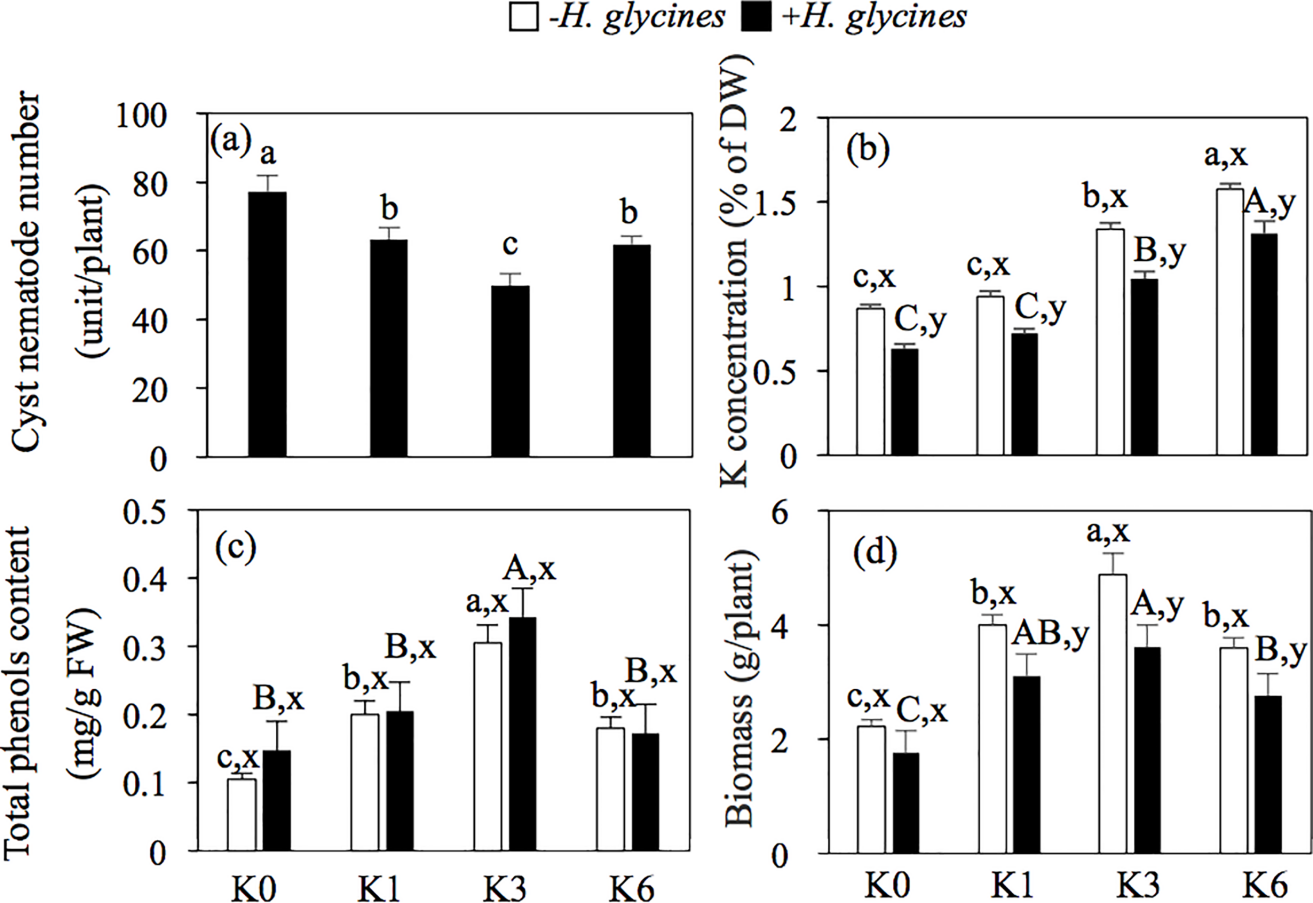
Effect of different K concentrations on cyst nematode number, K concentration, total phenol content and biomass of soybean in the sand culture. K0, No K application; K1, 1 mM K; K3, 3 mM K; K6, 6 mM K; +*H*. *glycines* means inoculation with *Heterodera glycines*; -*H*. *glycines* means no inoculation with *Heterodera glycines*. Each histogram represents the mean of four replicates ± SE. Different upper-case letters (A, B, C) above the bars indicate significant differences (*P<0*.*05*) by Duncan’s t-tests on inoculation with *H*. *glycines* treatment. Lower-case letters (a, b, c) mean no inoculation with *H*. *glycines* treatment. x,y indicate the different inoculation treatments within the same K concentration treatment. The same designations are used below.

In order to explore the relationship between infection of *H*. *glycines* and the application of different K concentrations, sand cultures were conducted in the greenhouse. As shown in Fig 2A, the cyst nematode number was dramatically affected by the supply of K. Compared to the K0 treatment, all of the other treatments had significantly lower cyst nematode numbers. For example, application of K3 treatment resulted in the lowest soybean cyst nematode number, decreased by 35% compared to that of K0 treatment. An intermediate level of cyst numbers was obtained in soybean roots as the result of K1 and K6 treatments (Fig 2A). It was revealed that a suitable K concentration could effectively control SCN.

### Physiological index and biomass of soybean in sand culture

The biomass and K concentration of the soybean plants were negatively affected by infected *H*. *glycines*, but there was no significant difference in total phenol content (Fig 2). Furthermore, the highest quantitative value was determined under the K3 treatment as indicated by the biomass and content of total phenol either in inoculation or without inoculation (Fig 2C and 2D). However, at a 6 mM K concentration, the values of biomass and content of total phenol dropped. For example, the content of total phenol and biomass under the K6 treatment decreased by 16% and 42% compared to that under the K3 treatment without inoculation, respectively. Nevertheless, the K concentration of plants increased with the increase in K supply (Fig 2B).

### Expression of related resistant genes to disease and activities of enzymes in the sand culture

**Fig 3:**
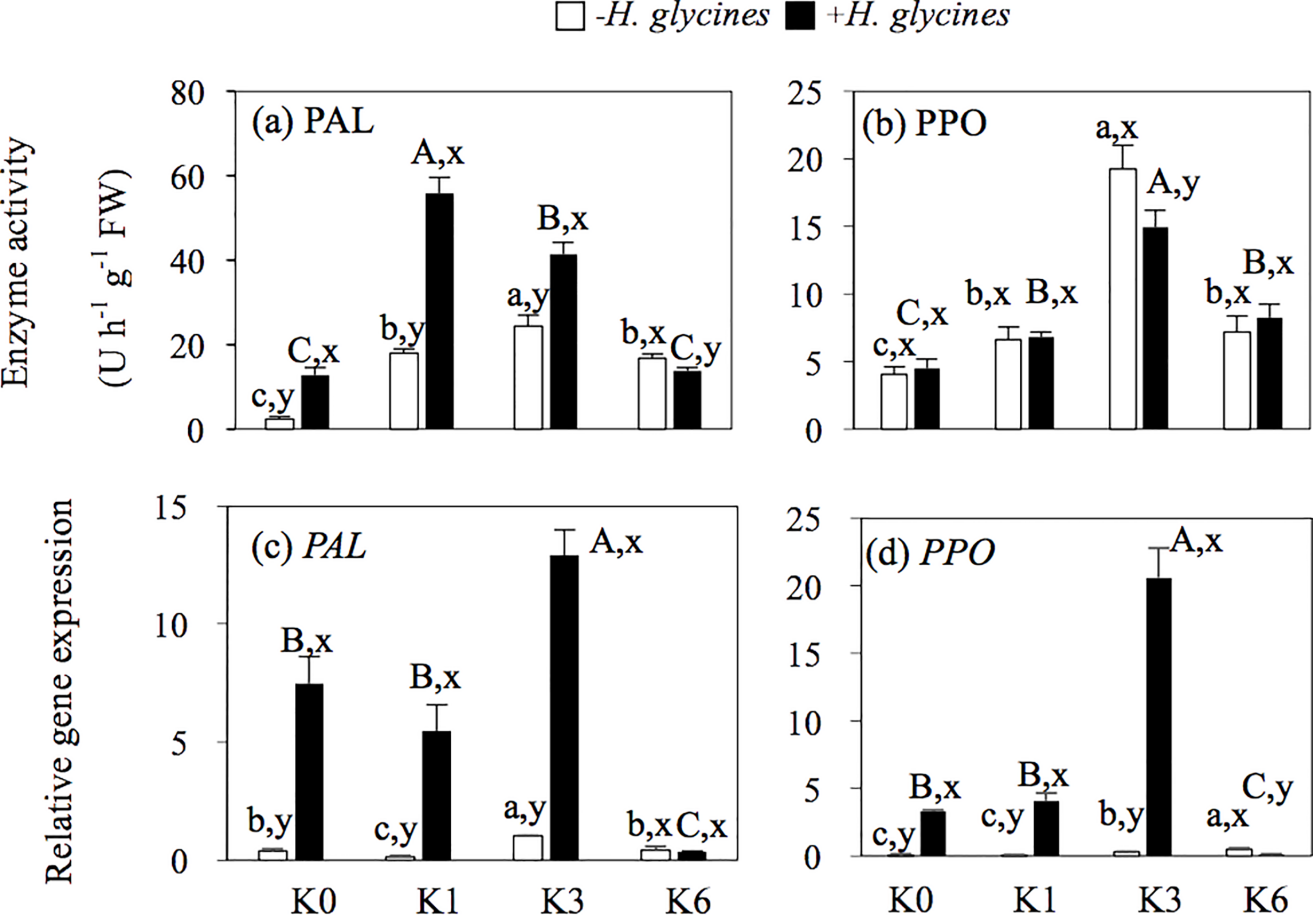
Expression of two defense-related genes and their enzyme activity in roots of soybean in response to *H*. *glycines* infection under the treatment of different K concentrations in the sand culture experiment. K0, No K application; K1, 1 mM K; K3, 3 mM K; K6, 6 mM K; +*H*. *glycines* means inoculation with *Heterodera glycines*; -*H*. *glycines* means no inoculation with *Heterodera glycines*.

The results in Fig 3 show that the enzyme activities of PAL and PPO in soybean roots were enhanced by K application whether inoculated by *H*. *glycines* or not (Fig 3A and 3B). The PAL activity under K1 treatment had the highest value in the inoculated *H*. *glycines* treatments and significantly increased 3.4-fold in comparison to K0. Of the non-inoculated *H*. *glycines* treatments, the K3 quantitative value was the highest, followed by K1 (Fig 3A). The PPO activity under K3 treatment had the highest value of both the inoculated and non-inoculated *H*. *glycines* treatments and significantly increased 3.7- and 2.2-fold compared to K0 treatment, respectively (Fig 3B). The transcript abundance of tested pathogen-related genes in soybean roots was altered by inoculation of *H*. *glycines* and was partly dependent on K levels (Fig 3C and 3D). The relative expression values of *PAL* and *PPO* reached their highest levels under the K3 treatments by inoculation of the *H*. *glycines* treatments and were significantly increased 1.7- and 5.1-fold compared to K0 treatment, respectively. However, under K6 treatments, the transcripts of the tested two genes exhibited their lowest expression in inoculated *H*. *glycines* treatments.

### The effects of K application on the phenolic acid release in root exudates

**Fig 4:**
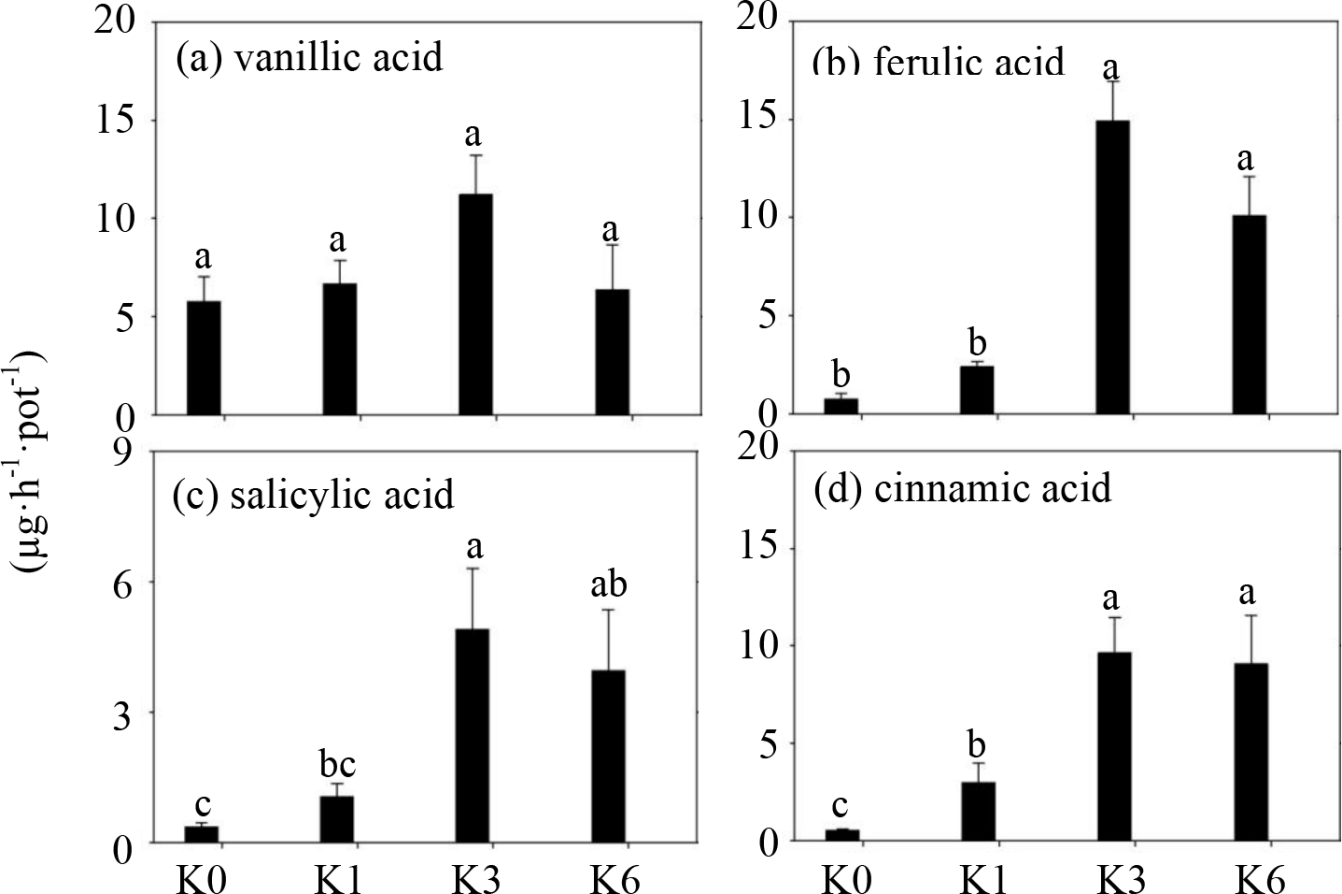
The exudation rate of four phenolic acids from soybean roots under treatments of different K concentrations in the sandy culture at the flowering stage. K0, No K application; K1, 1 mM K; K3, 3 mM K; K6, 6 mM K; Each histogram represents the mean of four replicates ± SE. Different letters above the bars indicate significant differences (*P<0*.*05*) by Duncan’s t-tests.

As shown in Fig 4, four phenolic acids, including vanillic, ferulic, cinnamic and salicylic acids, were detected in root exudates. The release rates of phenolic acids in root exudates were found to be variable and exhibited a strong correlation with the different K treatment levels. Under K3 treatment, greater amounts of various phenolic acids were released by roots (Fig 4). Compared to K0 and K1 treatments, the K3 treatment resulted in a greater release rate of cinnamic acid of 24.2- and 5.3-fold, respectively (Fig 4D). Furthermore, K3 treatment resulted in the highest content of salicylic acid that was 13- and 3.6-fold higher than the K0 and K1 treatments, respectively, while the content under K6 treatment decreased by 19% (Fig 4C). Ferulic acid showed the highest secretion rate under K3 treatment (Fig 4B). However, there was no significant difference detected in all of the treatments with different K concentrations for secretion of vanillic acid (Fig 4A).

### Effect of root exudates and exogenously applied phenolic acids on the mortality rate of *H*. *glycines in vitro*

**Fig 5:**
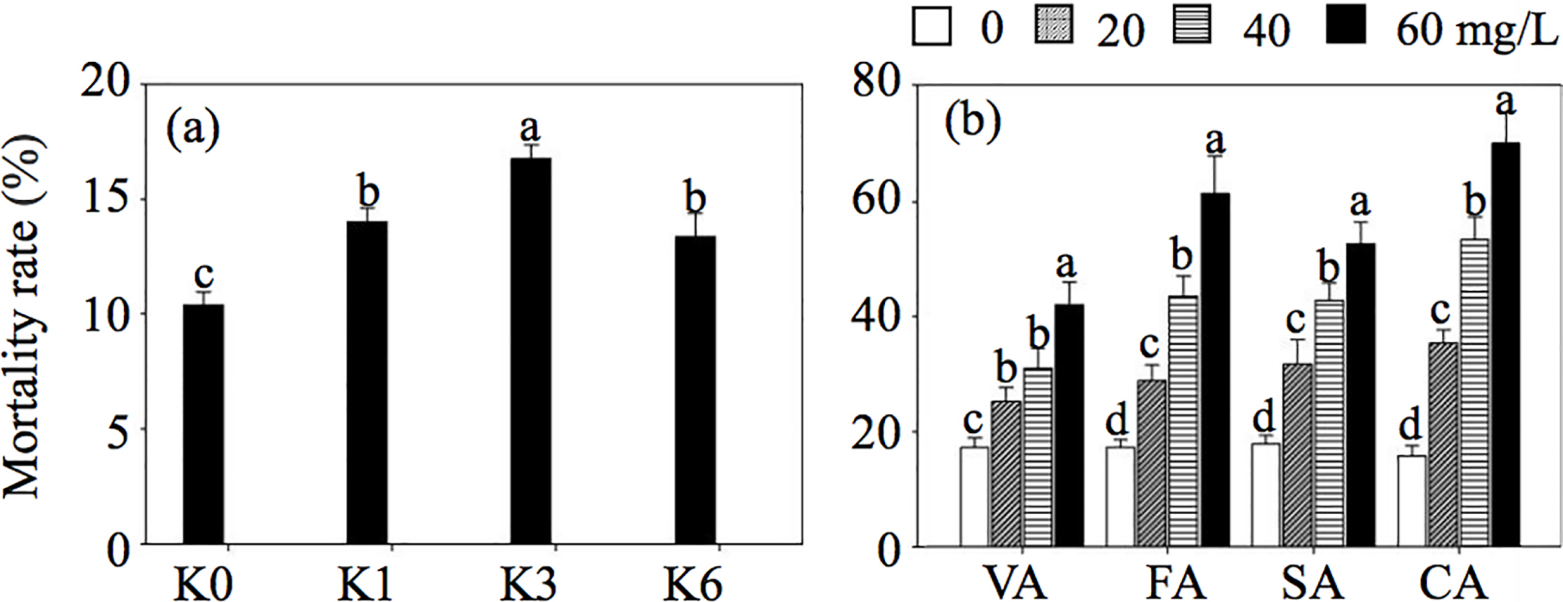
Effect of applied root exudates from soybean cultivated under different K concentration treatments (a) and of four different species of phenolic acids at four concentrations (b) on the mortality rate of *Heterodera glycines*. K0, No K application; K1, 1 mM K; K3, 3 mM K; K6, 6 mM K; VA is vanillic acid; FA is ferulic acid; SA is salicylic acid; CA is cinnamic acid. Each histogram represents the mean of four replicates ± SE. Different letters above the bars indicate significant differences (*P<0*.*05*) by Duncan’s t-tests.

In comparison with K0, the addition of root exudates from the K3 treatment dramatically improved SCN mortality rate (Fig 5A). Mortality of *H*. *glycines* increased by 38% compared to the K0 treatment. This result suggested that the adaptive K application could trigger the plant to secrete antagonistic substances and resist to pathogen activity.

Because soybean root exudates can alter the mortality of *H*. *glycines* under different K concentrations, the effect of exogenously applied individual phenolic acids on *H*. *glycines* activity was detected *in vitro*. Along with the root exudates, the addition of individual phenolic acids effectively increased the mortality rate of *H*. *glycines* compared to the control (Fig 5B). For example, the mortality of *H*. *glycines* had the highest rate under cinnamic acid treatment. When its concentration was 60 mg L^-1^, the mortality rate was 70%, an increase of 54% in comparison with 0 mg L^-1^. The same trend was found in the case of other phenolic acids, and mortality rates dramatically increased with an increase in the concentrations of individual phenolic acids.

## Discussion

Potassium is not only an essential plant nutrient, but it is also a modifier that can improve plant resistance against biotic and abiotic stresses [20–22]. However, most K fertilizer studies have focused on plant resistance performance in the field. Few plant studies have paid attention to inherent mechanisms, particularly in root exudation of phenolic acids and plant-related disease resistance genes. In this study, we demonstrated that application of an appropriate amount of K fertilizer induce the plant’s defense potential against SCN disease at both the physiological and molecular levels from field trials and greenhouse experiments.

The application of K fertilizer can enhance plant resistance against pathogen attack in most cases [12,23], probably due to sufficient K input enabling the plants to allocate more resources to develop more robust plants, which could prevent the invasion of phytopathogens [11,14]. In the current study, we observed that application of reasonable K fertilizer inputs significantly ameliorated the severity of SCN and the cyst numbers on rhizosphere systems based on two-year field trials (Table 1). Further sand culture experiments using different K concentration treatments confirmed the results (Fig 2). It is worth noting that when K fertilizer inputs reach 120 kg/ha, DI is not significantly reduced compared to the 60 kg/ha treatment in the field trials (Table 1). Similar results were found in sand culture when the K concentration exceeds 3 mM (Fig 2). This point is supported by reports that in order to control the disease effectively, the amount of fertilizer needs to be moderate but not excessive [16, 23]. Moreover, we also found that in the field and greenhouse experiments, the content of total phenol was obviously altered with the different K fertilizer inputs (Table 2 and Fig 2), and it is closely related to disease resistance [11,21].

Phenol compounds play an important role in enhancing a plant’s resistance to disease [20,24–26]. Potassium deficiency results in a reduction in the concentration of phenols, making plants more vulnerable to disease attack [11]. Accordingly, with an increase in the concentration of K fertilizer inputs, higher concentrations of K and phenol were found in the plants in both the field and greenhouse experiments (Table 2 and Fig 2). In addition, plants respond to pathogen attack by enhancing the synthesis of phenol compounds [11, 24]. We observed a higher level of phenol content with a suitable K application, which is in agreement with the fact that it is related to a plant’s resistance against *H*. *glycines*.

The root exudates are involved in creating an interaction between the roots and soil microorganisms and producing and secreting chemical substances into the rhizosphere to protect the plants from phytopathogens [27,28]. Meanwhile, allelochemical substances were significantly altering the root exudation patterns through different nutrient application [18,20]. In the sand culture experiment, it was found that the plants were more resistant against SCN attack under the K3 (3 mM K) treatment than under the other treatments through a reduction in the cyst number in the root systems to a large extent (Fig 2A), which means that the specific root exudates could inhibit phytopathogens. This is supported by *in vitro* assays where a significant increase in the mortality rate of *H*. *glycines* was detected as a result of exogenous application of individual root exudates (Fig 5A). Therefore, it is reasonable to speculate that adequate K inputs released more allelopathic compounds inhibiting *H*. *glycines* activity in the rhizosphere.

Phenolic acids secreted by plant roots have been considered to be the main allelochemicals, which can suppress the growth of phytopathogens in agricultural and ecological systems [18,19,29,30,31]. In order to detect phenolic acids that inhibit the activity of *H*. *glycines*, we investigated eight phenolic acids from the soybean roots under four K treatments. Four phenolic acids were identified from the soybean roots using HPLC (Fig 4). Among these four phenolic acids, the release of cinnamic acid was conspicuously higher under K3 treatment (Fig 4), which is thought to inhibit phytopathogens when exogenously applied alone, and it appears to be an allelochemical that remarkably restrains pathogen growth and germination [32]. Among these identified four phenolic acids, cinnamic acid was not only heavily released (Fig 4D) but also induced a higher mortality rate under K3 treatment than the other compounds *in vitro* (Fig 5B). Therefore, it is reasonable to deduce that cinnamic acid is one explanation for the protection of soybean roots from *H*. *glycines* attack when applying different K treatments. In this study, root exudates under K3 treatment produced more salicylic acid than that of no K inputs, suggesting that salicylic acid may contribute to the promotion of the plant’s resistance against disease invasion. Salicylic acid, as an allelochemical, may have inhibited pathogen activity and nematode population densities [32,33]. It was found that ferulic acid effectively increased the mortality rate of *H*. *glycines* (Fig 5B) as its content increased under the K3 treatment (Fig 4B). It has been reported to be an inhibitor of toxin production of pathogens [19,32]. Because plant roots can release a wide range of substances, more efficient phytotoxins from roots will hopefully be discovered in the future.

The results indicated that K could be a strong factor in priming PR protein inducible defense responses to disease (Fig 3). Many of the genes encoding PR proteins have been frequently demonstrated to be expressed in plants with infestation by pathogens and are involved in biosynthesis with a variety of phytoalexins to defend against biotic stress [24,34]. Consistent with this, the results herein show that defense responses are elicited by inoculation of *H*. *glycines* with reasonable K inputs in the roots of soybean. Analysis of PR defense associated with transcripts of genes and enzymes confirmed that it contained antimicrobial protein genes, such as PPO (polyphenol oxidase) and PAL (phenylalanine ammonia-lyase), which were greatly enhanced by the pathogen infestation and K amendment (Fig 3). When the plant is attacked by a pathogen, PPO and PAL can produce a number of chemical substances to fight against the disease, and different K inputs can also stimulate the resistance of the plant against phytopathogens [35,36]. Induction of PAL activity has been proved to initiate of the salicylic acid signaling pathways [17], and enhancement of phenolic compounds is in line with our results (Fig 4). The results revealed that the K inputs alone did not induce rapid expression of *PPO* and *PAL* at transcriptional levels. The *PPO* and *PAL* genes exhibited more rapid induction with transcriptional responses when *H*. *glycines* infestation and K application were combined (Fig 3). At the same time, the expression of PR was triggered by the K inputs with the highest up-regulation in response to K3 treatment except for the PAL enzyme activity under a non-inoculated pathogen treatment. Conversely, excessive K application decreased the PR at the transcriptional and enzyme levels (Fig 3). Thus, the results implied that transcripts of PR genes and enzyme activity were provoked by suitable K application, leading to increased soybean inherent defense potential against SCN invasion by lowering the risk of pathogenic infection.

In conclusion, this study demonstrates that the application of K fertilizer can effectively increase the plant’s inherent defense potential against SCN in both field trials and pot sand culture experiments, which increased the root exudation of phenolic acids and plant pathogen-related genes expression. The application of K fertilizer at an optimal concentration could have the strongest ability to inhibit *H*. *glycines* activity, whereas excessive K input does not appear to be helpful in SCN control. It is suggested that the application of optimal K fertilizer can be an effective tool to sustainably control soil-borne diseases. Thereby, it can improve crop quality and reduce the need for nematicide application and should be incorporated into economically and environmentally sustainable agriculture.

## Supporting information

S1 File The detailed calculation methods on PPO and PAL expression by RT-qPCR. (DOCX)

S2 File The minimal anonymized dataset for the tables and figures. (XLSX)

## References

[1] Maafi ZT, Salati M, Riggs RD. Distribution, population density, race and type determination of soybean cyst nematode, *Heterodera glycines*, in Iran. Nematology. 2008; 10: 919–924.

[2] Zhu Y, Tian J, Shi F, Su L, Liu K, Xiang M, et al Rhizosphere bacterial communities associated with healthy and *Heterodera glycines* -infected soybean roots. European Journal of Soil Biology. 2013; 58: 32–37.

[3] Liu S, Kandoth PK, Warren SD, Yeckel G, Heinz R, Alden J, et al A soybean cyst nematode resistance gene points to a new mechanism of plant resistance to pathogens. Nature. 2012; 492: 256–260. 10.1038/nature11651 23235880

[4] Wang J, Niblack TL, Tremain JA, Wiebold WJ, Tylka GL, Marett CC, et al Soybean cyst nematode reduces soybean yield without causing obvious aboveground symptoms. Plant Disease. 2003; 87: 623–628. 10.1094/PDIS.2003.87.6.623 30812850

[5] Niblack TL. Soybean cyst nematode management reconsidered. Plant Disease. 2005; 89: 1020–1026. 10.1094/PD-89-1020 30791267

[6] Ntalli NG, Caboni P. Botanical nematicides: a review. Journal of Agricultural and Food Chemistry. 2012; 60: 9929–9940. 10.1021/jf303107j 22973877

[7] Qiao K, Shi X, Wang H, Ji X, Wang K. Managing root-knot nematodes and weeds with 1,3-dichloropropene as an alternative to methyl bromide in cucumber crops in China. Journal of Agricultural and Food Chemistry. 2011; 59: 2362–2367. 10.1021/jf104553f 21366311

[8] Zhou Y, Wang Y, Zhu X, Liu R, Xiang P, Chen J, et al Management of the soybean cyst nematode *Heterodero glycines* with combinations of different rhizobacterial strains on soybean. Plos One. 2017, 8, e0182654. 10.1371/journal.pone.0182654 PMC5542665 28771591

[9] Chitwood DJ. Phytochemical based strategies for nematode control. Annual Review of Phytopathology. 2002; 40: 221–249. 10.1146/annurev.phyto.40.032602.130045 12147760

[10] Dordas C. Role of nutrients in controlling plant diseases in sustainable agriculture. A review. Agronomy for Sustainable Development. 2008; 28: 33–46.

[11] Wang M, Zheng Q, Shen Q, Guo S. The critical role of potassium in plant stress response. International Journal of Molecular Sciences. 2013; 14: 7370–7390. 10.3390/ijms14047370 23549270 PMC3645691

[12] Holzmueller EJ, Jose S, Jenkins MA. Influence of calcium, potassium, and magnesium on *Cornus florida* L. density and resistance to dogwood anthracnose. Plant and Soil. 2007; 290:189–199.

[13] Amtmann A, Troufflard S, Armengaud P. The effect of potassium nutrition on pest and disease resistance in plants. Physiologia Plantarum. 2008; 133: 682–691. 10.1111/j.1399-3054.2008.01075.x 18331404

[14] Zörb C, Senbayram M, Peiter E. Potassium in agriculture–status and perspectives. Journal of Plant Physiology. 2014; 171: 656–669. 10.1016/j.jplph.2013.08.008 24140002

[15] Li W, He P, Jin J. Potassium influenced phenylalanine ammonia-lyase, peroxidases and polyphenol oxidases in Fusarium graminearum infected maize (*Zea mays* L.). Proceedings of the International Plant Nutrition Colloquium XVI 2009.

[16] Gao X, Lu X, Wu M, Zhang H, Pan R, Tian J, et al Co-inoculation with rhizobia and AMF inhibited soybean red crown rot: from field study to plant defense-related gene expression analysis. Plos One. 2012; 7: e33977 10.1371/journal.pone.0033977 22442737 PMC3307780

[17] Song YY, Zeng RS, Xu JF, Li J, Shen X, Yihdego WG. Interplant communication of tomato plants through underground common mycorrhizal networks. Plos One. 2010; 5:e13324 10.1371/journal.pone.0013324 20967206 PMC2954164

[18] Gao X, Wu M, Xu R, Wang X, Pan R, Kim HJ, Liao H. Root interactions in a maize/soybean intercropping system control soybean soil-borne disease, red crown rot. Plos One. 2014; 9: e95031 10.1371/journal.pone.0095031 24810161 PMC4014482

[19] Ling N, Huang Q, Guo S, Shen Q. *Paenibacillus polymyxa* SQR-21 systemically affects root exudates of watermelon to decrease the conidial germination of *Fusarium oxysporum* f.sp. *niveum*. Plant and Soil. 2011; 341: 485–493.

[20] Li W, He P, Jin J. Effect of potassium on ultrastructure of maize stalk pith and young root and their relation to stalk rot resistance. Agricultural Sciences in China. 2010; 9: 1467–1474.

[21] Cheynier V, Comte G, Davies K, Lattanzio V and Martens S. Plant phenolics: recent advances on their biosynthesis genetics, and ecophysiology. Plant Physiology and Biochemistry. 2013; 72: 1–20. 10.1016/j.plaphy.2013.05.009 23774057

[22] Midiwo JO, Yenesew A, Juma BF, Derese S, Ayoo JA, Aluoch AO, et al Bioactive compounds from some Kenyan ethnomedicinal plants: *Myrsinaceae*, *Polygonaceae* and *Psiadia punctulata*. Phytochemistry Reviews. 2002; 1: 311–323.

[23] Huber DM and Graham RD. The role of nutrition in crop resistance and tolerance to diseases In: Rengel Z (Ed.), Mineral nutrition of crops fundamental mechanisms and implications. Food Product Press, New York, 1999; pp: 205–226.

[24] Shivashankar S, Sumathi M, Krishnakumar NK, Rao VK. Role of phenolic acids and enzymes of phenylpropanoid pathway in resistance of chayote fruit (*Sechium edule*) against infestation by melon fly, *Bactrocera cucurbitae*. Annals of Applied Biology. 2015; 166: 420–433.

[25] Dicko MH, Gruppen H, Barro C, Traore AS, Berkel WJ, Voragen AG. Impact of phenolic compounds and related enzymes in sorghum varieties for resistance and susceptibility to biotic and abiotic stresses. Journal of Chemical Ecology. 2005; 31: 2671–2688. 10.1007/s10886-005-7619-5 16273434

[26] Nicholson RL and Hammerschmidt R. Phenolic compounds and their role in disease resistance. Annual Review of Phytopathology. 1992; 30: 369–389.

[27] Ren L, Su S, Yang X, Xu Y, Huang Q, Shen Q. Intercropping with aerobic rice suppressed Fusarium wilt in watermelon. Soil Biology Biochemistry. 2008; 40: 834–844.

[28] Bais HP, Prithiviraj B, Jha AK, Ausubel FM, Vivanco JM. Mediation of pathogen resistance by exudation of antimicrobials from roots. Nature. 2011; 434: 217–221. 10.1038/nature03356 15759001

[29] Bais HP, Weir TL, Perry LG, Gilroy S, Vivanco JM. The role of root exudates in rhizosphere interactions with plants and other organisms. Annual Review of Plant Biology. 2006; 57: 233–266. 10.1146/annurev.arplant.57.032905.105159 16669762

[30] Shaukat SS, Siddiqui IA, Ali NI, Ali SA. Nematicidal and allelopathic responses of *Lantana camara* root extract. 2003; 42: 71–78.

[31] Wu HS, Raza W, Fan JQ, Sun YG, Bao W, Liu DY, et al Antibiotic effect of exogenously applied salicylic acid on in vitro soilborne pathogen, *Fusarium oxysporum* f.sp.*niveum*. Chemosphere. 2008; 74: 45–50. 10.1016/j.chemosphere.2008.09.027 18952255

[32] Hao WY, Ren LX, Ran W, Shen QR. Allelopathic effects of root exudates from watermelon and rice plants on *Fusarium oxysporum* f.sp. *niveum*. Plant and Soil. 2010; 336: 485–497.

[33] Wuyts N, Swennen R, De Waele D. Effects of plant phenylpropanoid pathway products and selected terpenoids and alkaloids on the plant parasitic nematodes *Radopholus similis*, *Pratylenchus penetrans* and *Meloidogyne incognita*. Nematology. 2006; 8: 89–101.

[34] van Loon LC, Rep M, Pieterse CM. Significance of inducible defense-related proteins in infected plants. Annual Review of Phytopathology. 2006; 44: 135–162. 10.1146/annurev.phyto.44.070505.143425 16602946

[35] Klink VP, Matthews BF. Emerging approaches to broaden resistance of soybean to soybean cyst nematode as supported by gene expression studies. Plant Physiology. 2009; 151: 1017–1022. 10.1104/pp.109.144006 19675146 PMC2773110

[36] Upchurch RG, Ramirez ME. Defense-related gene expression in soybean leaves and seeds inoculated with *Cercospora kikuchii* and *Diaporthe phaseolorum* var. *meridionalis*. Physiological and Molecular Plant Pathology. 2010; 75: 64–70.

