# Supplementary material for: Potassium-induced plant resistance against soybean cyst nematode via root exudation of phenolic acids and plant pathogen-related genes
Source: PLoS One. 2018 Jul 30;13(7):e0200903. doi: 10.1371/journal.pone.0200903 (PMC6066213; doi:10.1371/journal.pone.0200903)
Supplement: S1 File — (DOCX) [file pone.0200903.s001.docx]

The detailed calculation methods on PPO and PAL expression by RT-qPCR.

Soybean housekeeping genes (accession no.X56856):

TefS1-F, TGCAAAGGAGGCTGCTAACT

TefS1-R, CAGCATCACCGTTCTTCAAA

Standard curve:

PPO: R^2^=0.99


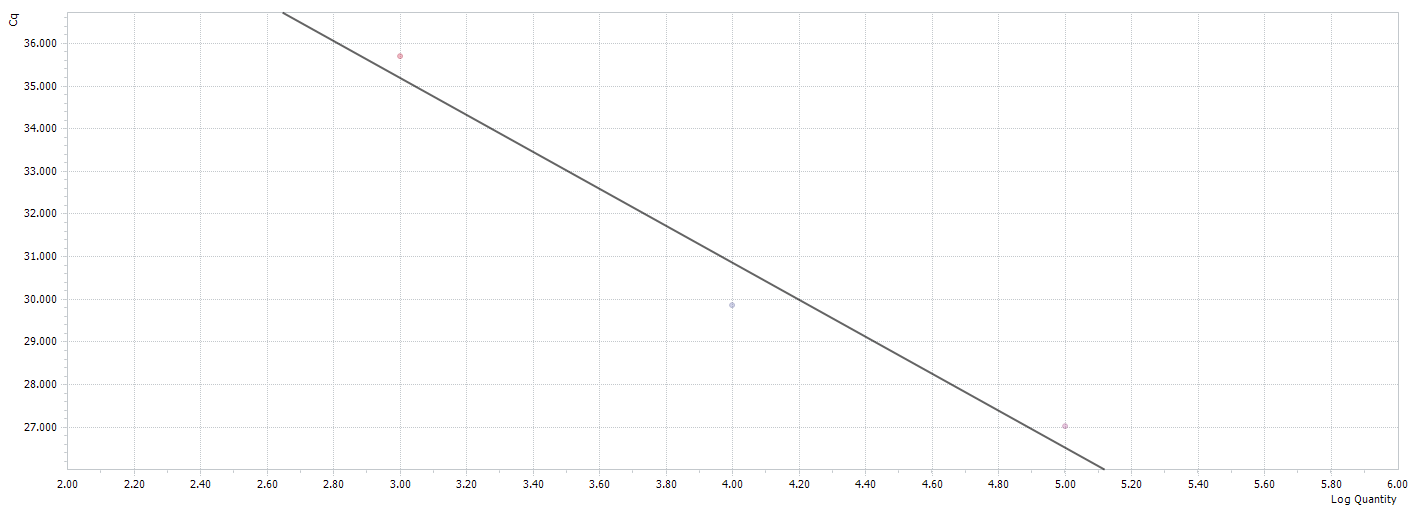


PAL: R^2^=0.96


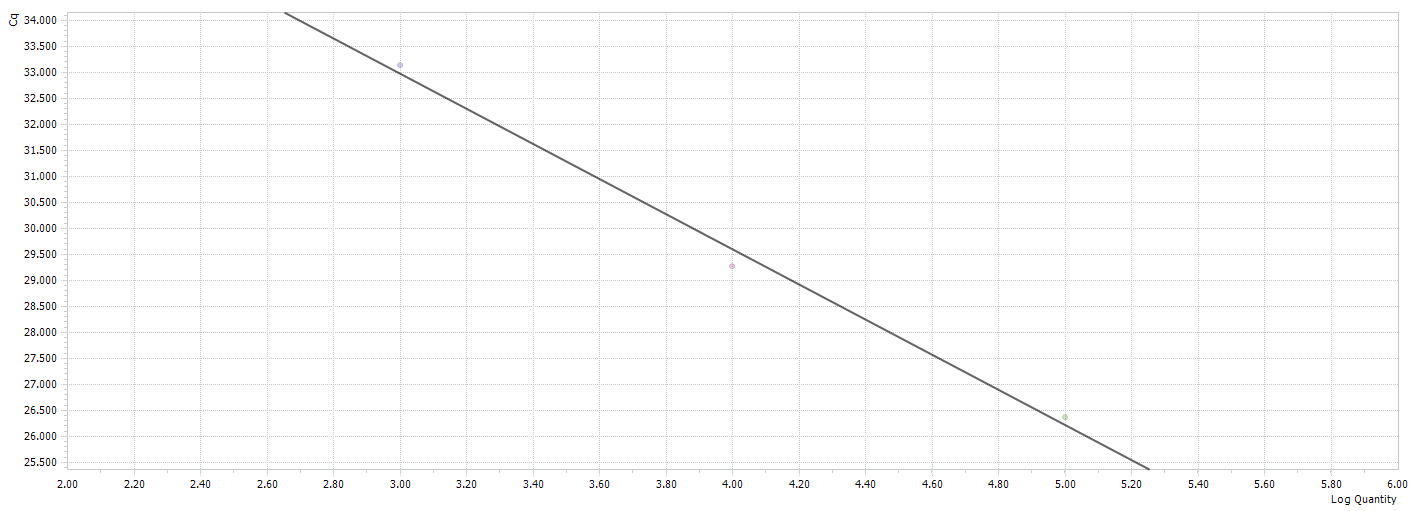


melting curve:

PPO:


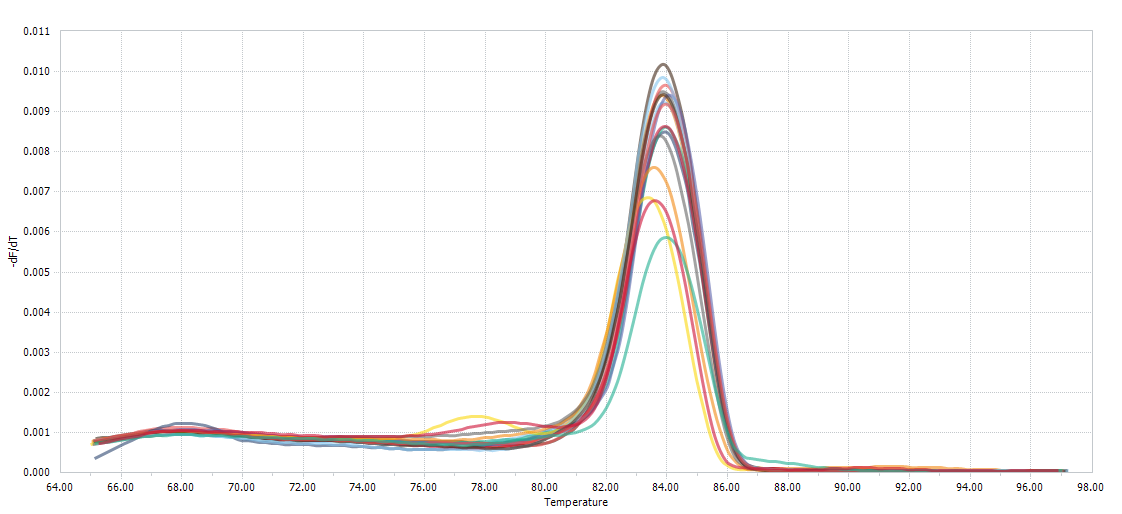


PAL:


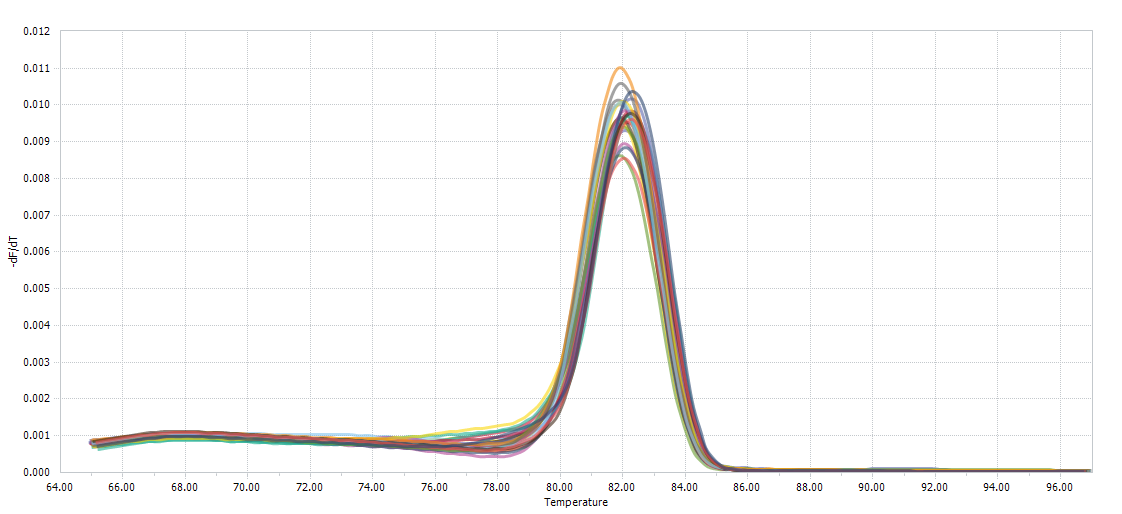


RAW DATA:

PAL relative expression (RE) = pal raw data/ TefS1 data

PPO relative expression(RE) = ppo raw data/ TefS1 data

| Treat. | TefS1  data | pal  Raw data | pal  RE | ppo  Raw data | ppo  RE |
| --- | --- | --- | --- | --- | --- |
| KO- | 160200 | 28570 | 0.178 | 1048 | 0.007 |
| KO- | 7737 | 4355 | 0.563 | 1059 | 0.137 |
| KO- | 4909 | 1790 | 0.365 | 247 | 0.050 |
| KO+ | 10020 | 95240 | 9.505 | 32490 | 3.243 |
| KO+ | 8533 | 62470 | 7.321 | 30000 | 3.516 |
| KO+ | 3558 | 20005 | 5.623 | 11442 | 3.216 |
| K1- | 82970 | 12460 | 0.150 | 5220 | 0.063 |
| K1- | 75760 | 4283 | 0.057 | 2706 | 0.036 |
| K1- | 29250 | 6619 | 0.226 | 1671 | 0.057 |
| K1+ | 63150 | 330660 | 5.236 | 330667 | 5.236 |
| K1+ | 16250 | 59070 | 3.635 | 52221 | 3.214 |
| K1+ | 34350 | 257980 | 7.510 | 132059 | 3.845 |
| K3- | 19220 | 17630 | 0.917 | 6661 | 0.347 |
| K3- | 17790 | 19260 | 1.083 | 5712 | 0.321 |
| K3- | 22890 | 23300 | 1.018 | 6521 | 0.285 |
| K3+ | 1625 | 23940 | 14.732 | 35170 | 21.643 |
| K3+ | 1290 | 14180 | 10.992 | 30650 | 23.760 |
| K3+ | 1237 | 16030 | 12.959 | 20270 | 16.386 |
| K6- | 24380 | 8689 | 0.356 | 10620 | 0.436 |
| K6- | 10450 | 7133 | 0.683 | 6486 | 0.621 |
| K6- | 22260 | 5217 | 0.234 | 10790 | 0.485 |
| K6+ | 44510 | 15070 | 0.339 | 5417 | 0.122 |
| K6+ | 36330 | 10440 | 0.287 | 5743 | 0.158 |
| K6+ | 32260 | 13070 | 0.405 | 4977 | 0.154 |

Gene relative expression summary:

| PAL gene | No |  |  | Means | SE |
| --- | --- | --- | --- | --- | --- |
| K0 | 0.178 | 0.563 | 0.365 | 0.37 | 0.11 |
| K1 | 0.150 | 0.057 | 0.226 | 0.14 | 0.05 |
| K3 | 0.917 | 1.083 | 1.018 | 1.01 | 0.05 |
| K6 | 0.356 | 0.683 | 0.234 | 0.42 | 0.13 |
|  | Inoculated |  |  |  |  |
| K0 | 9.505 | 7.321 | 5.623 | 7.48 | 1.12 |
| K1 | 5.236 | 3.635 | 7.510 | 5.46 | 1.13 |
| K3 | 14.732 | 10.992 | 12.959 | 12.89 | 1.08 |
| K6 | 0.339 | 0.287 | 0.405 | 0.34 | 0.03 |

| PPO gene | No |  |  | Means | SE |
| --- | --- | --- | --- | --- | --- |
| K0 | 0.007 | 0.137 | 0.050 | 0.06 | 0.04 |
| K1 | 0.063 | 0.036 | 0.057 | 0.05 | 0.01 |
| K3 | 0.347 | 0.321 | 0.285 | 0.32 | 0.02 |
| K6 | 0.436 | 0.621 | 0.485 | 0.51 | 0.06 |
|  | Inoculated |  |  |  |  |
| K0 | 3.243 | 3.516 | 3.216 | 3.32 | 0.10 |
| K1 | 5.236 | 3.214 | 3.845 | 4.10 | 0.60 |
| K3 | 21.643 | 23.760 | 16.386 | 20.60 | 2.19 |
| K6 | 0.122 | 0.158 | 0.154 | 0.14 | 0.01 |
